# Higher atypical enteropathogenic *Escherichia coli* (a-EPEC) bacterial loads in children with diarrhea are associated with PCR detection of the EHEC factor for adherence 1/lymphocyte inhibitory factor A (*efa1/lifa*) gene

**DOI:** 10.1186/s12941-017-0188-y

**Published:** 2017-03-23

**Authors:** Robert Slinger, Kimberley Lau, Michael Slinger, Ioana Moldovan, Francis Chan

**Affiliations:** 1 McMaster University, Hamilton, ON Canada; 2 Department of Laboratory Medicine and Pathology, Children’s Hospital of Eastern Ontario, University of Ottawa, 401 Smyth Rd, Ottawa, ON K1H 8L1 Canada

**Keywords:** Enteropathogenic *Escherichia coli*, Diarrhea, Real-time PCR, Gastroenteritis

## Abstract

**Background:**

Typical enteropathogenic *Escherichia coli* (t-EPEC) are known to cause diarrhea in children but it is uncertain whether atypical EPEC (a-EPEC) do, since a-EPEC lack the bundle-forming pilus (*bfp*) gene that encodes a key adherence factor in t-EPEC. In culture-based studies of a-EPEC, the presence of another adherence factor, called EHEC factor for adherence/lymphocyte activation inhibitor (*efa1/lifA)*, was strongly associated with diarrhea. Since a-EPEC culture is not feasible in clinical laboratories, we designed an *efa1/lifA* quantitative PCR assay and examined whether the presence of *efa1/lifA* was associated with higher a-EPEC bacterial loads in pediatric diarrheal stool samples.

**Methods:**

Fecal samples from children with diarrhea were tested by qPCR for EPEC (presence of e*ae* gene) and for shiga toxin genes to exclude enterohemorrhagic *E. coli,* which also contain the *eae* gene. EPEC containing samples were then tested for the bundle-forming pilus gene found in t-EPEC and *efa1/lifA*. The *eae* gene quantity in *efa1/lifA*-positive and negative samples was compared.

**Results:**

Thirty-nine of 320 (12%) fecal samples tested positive for EPEC and 38/39 (97%) contained a-EPEC. The *efa1/lifA* gene was detected in 16/38 (42%) a-EPEC samples. The median *eae* concentration for *efa1/lifA* positive samples was significantly higher than for *efa1/lifA* negative samples (median 16,745 vs. 1183 copies/µL, respectively, p = 0.006).

**Conclusions:**

Atypical enteropathogenic *E. coli*-positive diarrheal stool samples containing the *efa1/lifA* gene had significantly higher bacterial loads than samples lacking this gene. This supports the idea that *efa1/lifA* contributes to diarrheal pathogenesis and suggests that, in EPEC-positive samples, *efa/lifA* may be a useful additional molecular biomarker.

## Background

Enteropathogenic *Escherichia coli* (EPEC) is not detected with the standard stool culture methods used in clinical laboratories for bacterial pathogens, so its relative importance as a cause of diarrhea in children has been uncertain in the past [1]. With the recent increased use of molecular detection methods, however, EPEC has been found to be the most frequently detected bacterium in patients with diarrhea in developed countries [2, 3].

However, not all EPEC strains have the same ability to cause diarrhea. All EPEC and enterohemorrhagic *E. coli* (EHEC) contain the chromosomal *E. coli* attaching and effacing (*eae*) gene that encodes an outer membrane protein called intimin. Intimin mediates attachment to epithelial cells and leads to the attaching and effacing phenotype. EPEC can be differentiated from EHEC by the absence of the shiga toxin (*stx*) genes that are found in EHEC. EPEC is further classified into typical and atypical strains based on the presence or absence of the *E. coli* adherence factor containing the bundle-forming pilus (*bfp*) gene. EPEC containing the *bfp* gene are classified as typical EPEC (t-EPEC) [1]. Strains lacking *bfp* are classified as atypical EPEC (a-EPEC).

Typical enteropathogenic *E. coli* is accepted as a diarrhea pathogen, but the pathogenicity of a-EPEC is controversial. Some studies have shown an association with a-EPEC and diarrhea while others have not. In addition, a-EPEC can be found in asymptomatic children [4–6]. Diarrhea was seen in volunteers who ingested a-EPEC but to a lesser degree than in those who ingested t-EPEC [7, 8]. This controversy surrounding a-EPEC virulence has led to a search for additional gene markers in a-EPEC that might indicate pathogenicity. The *efa1/lifA* gene appears to be the leading virulence candidate. *Efa1/lifA* encodes for a large 385 kDa adhesion protein, called the EHEC factor for adherence (*Efa1*) since it was first described in an EHEC 0157:H7 strain [9]. *Efa1* was found to be identical to the lymphocyte inhibitory factor A protein (*lifA*) gene that lymphostatin, which inhibits lymphocyte proliferation and lymphokine production. The designation *efa1/lifA* was therefore given to the gene.

In study of 182 possible virulence markers in a-EPEC cultured strains from Norwegian children, the *efa1/lifA* gene had the strongest statistical association with diarrhea [9]. *Efa1/lifA* was present in 30% of a-EPEC strains from children with diarrhea and no strains from children without diarrhea (p = 0.0008). In a later study from Japan, *efa1/lifA* was detected in 33% of a-EPEC strains in individuals with diarrhea and 13% of those in a healthy control group [10]. This difference was statistically significant (p < 0.05), and *efa1/lifA* was the only gene examined which was significantly associated with diarrhea.

These studies demonstrating the importance of *efa1/lifA* were performed using stool cultures for EPEC, but this is a labor-intensive process that is not feasible in clinical microbiology laboratories, since it requires isolating multiple *E. coli* colonies in each specimen and then testing these individually for the genes of interest. We therefore developed a direct fecal *efa1/lifA* real-time quantitative PCR (qPCR) method to determine what proportion of fecal specimens containing a-EPEC also contained *efa1/lifA*. We then examined whether a-EPEC bacterial loads were higher in children when *efa1/lifA*-containing a-EPEC was present. Our hypothesis was that since the quantity of EPEC in fecal samples as measured by qPCR is associated with disease severity, infection with *efa1/lifA*-containing strains of a-EPEC might lead to higher bacterial loads [11].

Of note, work to clarify the pathogenicity of a-EPEC has become more crucial in recent years since EPEC are now detected by a commercially available molecular diarrheal panel, the Biofire FilmArray gastrointestinal panel (Biomerieux, Durham, NC) [2, 3]. This panel detects the *eae* gene, but not *bfp* or *efa1/lifA*. Since a-EPEC is much more likely than t-EPEC to be present in developed countries, clinicians seeing children with diarrhea whose samples test positive for EPEC will face uncertainty as to whether the organism detected is the cause of the illness. Additional molecular biomarkers that are associated with a-EPEC pathogenicity rather than the presence of the *eae* gene alone may therefore be useful.

## Methods

### Study site and ethics approval

This was a prospective observational study performed in the months June–Aug 2010–2013 at the Children’s Hospital of Eastern Ontario, Ottawa, ON Canada, a 165 bed tertiary care hospital serving a catchment area of 1.5 million. It was decided to collect samples in the summer months since a seasonal predominance increasing in these months has been described for both EPEC and EHEC [1, 5].

Ethics approval was obtained for the study from the hospital Research Ethics Board (CHEOREB # 12/194X). Residual aliquots of fecal samples submitted for bacterial stool culture from patients with diarrhea that would otherwise have been discarded were tested by PCR for the target bacterial genes described below. All samples submitted to the laboratory for bacterial stool culture over the study period were included in the study.

The results of bacterial stool culture were recorded. Fecal samples were saved at −80 °C until nucleic acid extraction was performed. All patients were ≤18 years of age. According to the ethics approval received, in order to ensure patient anonymity, we did not record patient age or gender.

### Outcome measures

Our main outcome measure was the difference, if any, in a-EPEC bacterial load in *efa1/lifA* positive vs. negative samples. The secondary outcome measures were the prevalence of a-EPEC and t-EPEC in fecal samples; the prevalence of *efa1/lifA* in samples in which a-EPEC was detected; and a comparison of EPEC prevalence to the prevalence of bacterial pathogens detected by culture.

## Laboratory methods

### Culture methods

Fecal samples were collected in Cary-Blair enteric transport medium. Samples were inoculated onto Blood agar plate, MacConkey agar plate, Hektoen enteric agar plate, Sorbitol MacConkey agar plate, *Campylobacter* agar plate and Selenite broth. The *Campylobacter* plate was placed in a microaerophilic environment at 42 °C. Other media were incubated in ambient air at 35 °C. After overnight incubation, the selenite broth was subcultured onto a Hektoen plate. Plates were examined for *Salmonella* species (spp.), *Shigella* spp., *Campylobacter* spp.*, Yersinia enterocolitica, E. coli* 0157:H7*, Aeromonas* spp*. Plesiomonas shigelloides* and *Vibrio* spp. Possible pathogens were identified using standard laboratory methods [12].

### PCR methods

DNA was extracted from fecal specimens using automated device (iPrep, Life Technologies, Carlsbad, CA). Extracted DNA was then treated to remove fecal PCR inhibitors using a commercial method (Zymo-Spin™ IV-µHRC Spin Filter Zymo Research, Irvine, CA).

Primer and 5′ exonuclease probe sequences for the study assays (*eae*, *stx*1, *stx*2, *bfp* and *efa1/lifA)* are shown in Table 1. The *efa1/lifA* assay was designed for this study while other assays had been previously published. *Efa1/lifA* assay design was performed using a commercial qPCR program (Allele ID, Premier Biosoft, Palo Alto CA). The limit of detection of the assay was measured by duplicate testing of 10-fold serial dilutions of a synthetic oligonucleotide target sequence (Ultramer, IDT, Coralville, IA).

**Table 1:** Gene targets PCR primer and probe sequences

Target gene/symbol	Forward primer	Reverse primer	Probe	Reference
Intimin (*eae*)	cattgatcaggatttttctggtgata	ctcatgcggaaatagccgtta	atagtctcgccagtattcgccaccaatacc	[11]
Shiga toxin 1(*stx*1)	gtggcattaatactgaattgtcatca	gcgtaatcccacggactcttc	tctgccggacacatag (MGB)	[12]^a^
Shiga toxin 2 (*stx*2)	gggcagttattttgctgtgga	tgttgccgtattaacgaaccc	ctatcaggcgcgttttgaccatcttcg	[13]
Bundle-forming pilus (*bfp*)	gcatcattccgttgttgg	ggaccatgtattatcaaaaacctg	ccgccttctgacaagctgtgttgg	[14]
EHEC factor for adherence 1/lymphocyte inhibitory factor A (*efa1/lifA*)	tcacaccagaattattacgtcacaca	atggtagtcaggtatacatccgtatttc	accggcacaatactccagactccagaaga	This study

*MGB* minor groove binder

^a^Modified from published

Probe and primer specificity were checked using Basic Local Alignment Search Tool (BLAST) sequence searches and by testing the assay against the following bacterial organisms: *Streptococcus pneumoniae* American type culture collection (ATCC) 49,619, *Streptococcus salivarius* ATCC 13,419 *Escherichia coli* ATCC 25,922, *Haemophilus influenzae* ATCC 49,766, *H. influenzae* ATCC 49,247, *H. parainfluenzae* ATCC 7901, *Klebsiella pneumoniae* ATCC 700,603, *Moraxella catarrhalis* ATCC 25,238, *Staphylococcus aureus* ATCC 29,247, *Neisseria gonorrhoeae* ATCC 49,226, *N. lactamica* ATCC 23,970, *Pseudomonas aeruginosa* ATCC 27,853, *Enterococcus faecalis* ATCC 29,212, *S. dysgalactiae* subsp. *equisimilis, S. agalactiae* (Group B Streptococcus), *S. intermedius, S. constellatus,* and *S. anginosus.*

QPCR was performed on all samples for the *eae* gene and for the *stx*-1 and *stx*-2 genes to exclude EHEC [12, 14, 15]. *Eae*-positive; *stx*-negative samples were then tested for the *bfp* gene to differentiate t-EPEC and a-EPEC and for the *efa1/lifA* gene. Probes were of the 5-prime exonuclease type and were labelled with fluorescein amidite. Four probes were obtained from IDT (Coralville, IA) and contained an internal quencher as well as a 3-prime end quencher. One probe contained a minor groove binder and no internal quencher and was obtained from Life Technologies (Carlsbad, CA).

PCR assays were prepared in 20 µL volumes in 96-well qPCR plate. Positive and negative controls (no template) were performed with each qPCR plate run. QPCR plates were covered with MicroAmp^®^ Optical Adhesive Film (Life Technologies Carlsbad, CA) to prevent cross-contamination. QPCR was performed with a 96 well fast cycling block on a ViiA7 thermocyler (Life Technologies) using 40 cycles of 2-temperature thermocyling (95 °C × 3 s and 60 °C × 30 s). QPCR was considered positive for the *eae*, *stx*1, *stx*2, and *efa1/lifA* genes if the cycle threshold value was ≤35 cycles and for the *bfp* gene if the cycle threshold value was ≤30 cycles. Specimens were classified as containing EPEC if they were *eae*- positive and *stx*-negative, and then as t-EPEC (*bfp*-positive) or a-EPEC (*bfp*-negative). A-EPEC samples were further classified as being *efa1/lifA*-positive or negative.

## Results

The limit of detection for the *efa1/lifA* assay was approximately 6 copies/PCR reaction. In comparison, the detection limit for the *eae* assay was one log higher at 60 copies/reaction. There was no cross-reactivity observed for the *efa1/lifA* assay with the non-*E. coli* bacterial species tested.

Three-hundred twenty fecal diarrheal samples were tested. The PCR and culture results are shown in Table 2. Thirty-nine of 320 (12%) fecal samples tested were found to have the EPEC marker profile (*eae*-positive, *stx*-negative). Of these EPEC samples, 38/39 (97%) were a-EPEC (*bfp*-negative), with only one t-EPEC (*bfp*-positive).

**Table 2:** PCR and culture results from diarrheal fecal samples

PCR	No. detected (%) n = 320
*Eae* positive	44 (14)
EPEC: *eae* positive/*stx*1 and *stx*2 negative	39 (12)
Atypical EPEC: *eae* positive/stx1 and *stx*2 negative/*bfp* negative	38 (12)
Typical EPEC: *eae* positive/*stx*1 and *stx*2 negative/*bfp* positive	1 (0.3)
EHEC: *eae* positive/*stx*1 or *stx*2 positive	5 (1.5)
*eae* positive/*stx* negative/*efa1/lifA* positive/*bfp* negative	16 (5)
*eae* positive/*stx* negative/*efa1/lifA* negative/*bfp* negative	22 (7)
Culture	
*Salmonella* spp.	13 (4)
*Campylobacter* spp.	5 (1.5)
*Shigella* spp.	3 (0.9)
*Plesiomonas shigelloides*	3 (0.9)
*Yersinia enterocolitica*	2 (0.6)
*E. coli* 0157:H7	1 (0.3)
*Aeromonas* spp.	1 (0.3)

*Eae*, *E. coli* attaching and effacing gene; *EPEC*, enteropathogenic *E. coli*; *stx*1, shigatoxin 1 gene; *stx*2, shigatoxin 2 gene; *bfp*, bundle-forming pilus gene; *EHEC,* enterohemorrhagic *E. coli*; *efa1/lifA*, EHEC factor for adherence 1/lymphocyte inhibitory factor A gene

The *efa1/lifA* gene was detected in 16/38 (42%) a-EPEC samples. The median *eae* concentration in these samples was 16,745 copies/µL (range 26–3152,879 copies/µL). For the 22 *efa1/lifA*-negative a-EPEC samples, the median *eae* concentration was 1183 copies/µL (range 9–338,770 copies/µL). The difference in median copies/µL between the two groups was statistically significant (p = 0.006, Wilcoxon two-sample test two-sided).

Bacterial cultures were positive in 27/320 samples (8.4%), with 28 organisms detected in total. Other bacterial pathogens were detected in 4/38 (10%) a-EPEC-positive samples. The most common single bacterial type identified by culture was *Salmonella* spp., found in 13/320 (4%) samples. Only 1 EHEC (an *E. coli* O157:H7) was detected by culture, while one or both *stx* genes were detected by qPCR in five samples, including the one culture-positive sample.

## Discussion

Our findings show that EPEC genes were present with relatively high frequency in fecal samples submitted for bacterial culture from children with diarrhea in Ontario, Canada. EPEC genes were detected in more samples than the most prevalent pathogen detected by stool culture, *Salmonella* spp. (12 vs. 4%, respectively). This relatively high rate of detection of EPEC in developed country settings when molecular testing is used has also been reported by others. For example, in a study of daycare attendees in the Netherlands using a qPCR method, EPEC was detected in 19.9% of stool samples [6]. Investigators using the Biofire Film Array GI panel (biomerieux) reported detecting eae in 15% of diarrheal samples in a European multicenter study and 29.49% of samples in a US study [2, 3]. The proportions of t-EPEC and a-EPEC were not stated in either study (the *bfp* gene is not included in the Biofire panel), but most are likely to have been a-EPEC, given the low prevalence of t-EPEC in developed countries [1].

However, as discussed previously, it remains uncertain whether a-EPEC caused the diarrhea seen in these patients. Other pathogens may have been present in our patients that were not detected by bacterial stool cultures performed. It is also possible that a-EPEC may be able to grow better in the diarrheic environment created by other organisms, but is not contributing to disease.

Volunteer studies have been performed to examine a-EPEC pathogenicity but the results of these studies were not conclusive. For example, diarrhea occurred in 9/10 volunteers who ingested a t-EPEC strain, but in 6/11 who ingested an a-EPEC strain [7]. In a second study, 11/13 adults developed diarrhea with t-EPEC ingestion, as compared to 5/30 who ingested *bfp*-negative mutant strains. Thus, strains lacking *bfp* show reduced virulence in adults [8]. However, interpreting these studies in the pediatric setting is difficult since these adult volunteers may have been exposed to EPEC strains earlier in life and developed some degree of immunity prior to the challenge study.

More recently, a strong statistical association between the presence of *efa1/lifA*-positive a-EPEC and the presence of diarrhea was noted in two studies, suggesting *efa1/lifA*-positive strains may be true diarrhea pathogens [9, 10]. Some research has been done that provides a biological basis for the contribution of *efa1/lifA* to a-EPEC pathogenicity. *Efa1/lifA* is known to be an adherence factor and, in a study by Badea, an *efa1/lifA* mutant EPEC was found to be significantly less adherent to epithelial cells than the parent *efa1/lifA*-positive strain. Additionally, human and rabbit hosts infected with an attaching and effacing (A/E) pathogens were found to produce antibodies to efa1 protein, and anti-efa1 antibodies reduced the adherence of *efa*-positive EPEC to epithelial cells [16]. These findings suggest that the *efa1/lifA* gene, in concert with the *eae* gene, may play a role in adherence of a-EPEC infections that could then lead to diarrhea.

We also observed that samples with *efa1/lifA* contained higher loads of a-EPEC (as measured by *eae* gene quantitation) than *efa1/lifA*-negative samples. Higher bacterial loads have been associated with occurrence of diarrhea in patients with a-EPEC, so our finding of this association between bacterial load and the presence of *efa1/lifA* suggests strains with *efa1/lifA* may be more pathogenic.

The source of a-EPEC detected in the gastrointestinal tract of children in our center is unknown. Interestingly, we detected over five times more samples with EPEC markers than EHEC markers (27 vs. 5), suggesting that children are exposed to a-EPEC than EHEC much more frequently in our region. A recent study reported that a-EPEC strains that have been associated with diarrhea were found frequently in chicken and chicken products, so it is possible that these foods may be one source of exposure [17].

There are several limitations to this pilot study that should be mentioned. First, samples from children without diarrhea were not tested, so the prevalence of a-EPEC or *efa1/lifA* in asymptomatic children in our region is unknown. As well, we studied samples collected over summer months, rather than year-round, so it is possible that the frequency of EPEC detection may have been different with sampling over the entire year. Our study also assumes that the genes detected by PCR belong to the same stool bacterium, rather than different bacterial strains. For example, stool samples positive for *eae* and *efa1/lifA* were assumed to contain an EPEC strain that possesses both genes. Another limitation is that PCR cannot differentiate live from dead bacteria. Thus, the detection EPEC genes could be due to dead bacteria that may have been ingested but perhaps killed during cooking. However, since methods to readily detect EPEC in culture in clinical laboratories are not available, molecular methods like PCR will likely be used as the method of choice for EPEC detection in clinical specimens, despite this drawback. Finally, we did not test fecal samples for viral or parasitic pathogens or for *Clostridium difficile.* Some children with EPEC detected may have had one of these micro-organisms in their fecal samples, which could suggest that EPEC may not have been the cause of the diarrhea in these cases. In future work, we plan to perform testing for these additional organisms as well as EPEC.

Our objective in the study was to assess whether *efa1/lifA* could be directly detected by qPCR in stool samples, and whether *efa1/lifA* status was related to bacterial load. We now hope to be able to perform a qPCR-based case–control study for a-EPEC and *efa1/lifA* that will deal with the limitations noted above.

## Conclusion

Given our findings and previous reports regarding the significance of *efa1/lifA,* continued research into the role this gene plays in a-EPEC infection is needed. New volunteer studies comparing diarrheal symptoms in those ingesting *efa1/lifA*-positive and *efa1/lifA*-negative a-EPEC would be helpful. As noted, we hope to perform a case–control study in which *eae* and *efa1/lifA* will be examined and quantified by direct fecal qPCR in children with diarrhea and healthy controls.

As pointed out in a recent review, there are many other unanswered questions regarding EPEC [18], including the pathogenicity of a-EPEC and the optimum antibiotic treatment, if any, for patients with diarrhea in whom EPEC is detected. Historically, t-EPEC diarrhea in infants has been treated with a variety of oral antibiotics, such as gentamicin and colistin, with reported success [19] but randomized controlled trials of antibiotic treatment have not been performed. For a-EPEC, no information is available regarding antibiotic treatment. Given the ongoing shift in clinical microbiology laboratories from culture methods that do not detect EPEC to molecular detection panels that do, there is a pressing need to address these questions to help guide patient care.

## Abbreviations

EPEC: enteropathogenic *Escherichia coli*
t-EPEC: typical EPEC
a-EPEC: atypical EPEC
EHEC: enterohemorrhagic *E. coli*
*eae*: *E. coli* attaching and effacing
*stx*: shigatoxin
*bfp*: bundle-forming pilus
*efa1/lifA*: EHEC factor for adherence/lymphocyte inhibitory factor A
qPCR: real-time quantitative PCR
species: spp.
